# Skin bacterial communities of neotropical treefrogs vary with local environmental conditions at the time of sampling

**DOI:** 10.7717/peerj.7044

**Published:** 2019-06-21

**Authors:** Angie Estrada, Myra C. Hughey, Daniel Medina, Eria A. Rebollar, Jenifer B. Walke, Reid N. Harris, Lisa K. Belden

**Affiliations:** 1 Department of Biological Sciences, Virginia Polytechnic Institute and State University (Virginia Tech), Blacksburg, VA, USA; 2 Centro de Ciencias Genómicas, Universidad Nacional Autónoma de México, Cuernavaca, Morelos, México; 3 Department of Biology, James Madison University, Harrisonburg, VA, USA; 4 Smithsonian Tropical Research Institute, Balboa, Ancón, Panamá, Panamá

**Keywords:** Amphibian skin microbiome, Rainfall, Neotropics, Temporal scale, *Agalychnis callidryas*, *Dendropsophus ebraccatus*, Lowlands, Temperature

## Abstract

The amphibian skin microbiome has been the focus of recent studies aiming to better understand the role of these microbial symbionts in host defense against disease. However, host-associated microbial communities are complex and dynamic, and changes in their composition and structure can influence their function. Understanding temporal variation of bacterial communities on amphibian skin is critical for establishing baselines from which to improve the development of mitigation techniques based on probiotic therapy and provides long-term host protection in a changing environment. Here, we investigated whether microbial communities on amphibian skin change over time at a single site. To examine this, we collected skin swabs from two pond-breeding species of treefrogs, *Agalychnis callidryas* and *Dendropsophus ebraccatus,* over 4 years at a single lowland tropical pond in Panamá. Relative abundance of operational taxonomic units (OTUs) based on 16S rRNA gene amplicon sequencing was used to determine bacterial community diversity on the skin of both treefrog species. We found significant variation in bacterial community structure across long and short-term time scales. Skin bacterial communities differed across years on both species and between seasons and sampling days only in *D. ebraccatus*. Importantly, bacterial community structures across days were as variable as year level comparisons. The differences in bacterial community were driven primarily by differences in relative abundance of key OTUs and explained by rainfall at the time of sampling. These findings suggest that skin-associated microbiomes are highly variable across time, and that for tropical lowland sites, rainfall is a good predictor of variability. However, more research is necessary to elucidate the significance of temporal variation in bacterial skin communities and their maintenance for amphibian conservation efforts.

## Introduction

Host-associated microbial communities promote health and fitness for animal and plant hosts alike. Although a healthy microbiome is yet to be defined, assessing the stability of microbial communities, their impact on host fitness and their response to disturbances at different spatio-temporal scales have become fundamental issues in microbial ecology (Antwis et al., 2017). However, measuring natural temporal variation in the microbiome can be daunting, despite emergent technologies for DNA sequencing and advanced computational tools (Arnold, Roach & Azcarate-Peril, 2016). Challenges include determining the necessary frequency of sampling, detecting, measuring and gauging the impact of ecosystem disturbance and tackling difficulties in the analysis of large time-series data (Gerber, 2014). Additionally, the environmental scales over which community change occurs still need to be determined (Shade et al., 2013). The latter represents one of the greatest challenges in the study of symbionts associated with free-living wildlife hosts. Depending on the host species and habitat type, survey efforts and physical monitoring may lack the temporal resolution to capture rapid community changes (Shade et al., 2013) and to disentangle the effect of environmental variables on the microbiome.

The relevance of temporal scale on variation of host-associated microbial communities has been mostly studied in human and model organisms (reviewed by Robinson, Bohannan & Young, 2010). In free-living wildlife, studies are limited, but, for example, recent field surveys combined with transplant experiments in wild Pacific oysters revealed temporal stability of their microbiome maintained across months (Lokmer et al., 2016). Amphibian studies generally suggest that longitudinal changes in skin microbiome occur during life stage transitions, especially from larvae to adults (Kueneman et al., 2014; Longo et al., 2015; Sabino-Pinto et al., 2017) and in adults, with transitions from aquatic to terrestrial environments (Bletz et al., 2017). These transitions in microbial community composition are likely correlated with marked shifts in environmental factors associated with the life stages or microhabitats. As in other ectotherms (e.g., lizards, Bestion et al., 2017), seasonal shifts in temperature are of the strongest drivers known to impact amphibian-associated microbial communities, having been observed in both tadpoles (Kohl & Yahn, 2016) and adults (Bletz et al., 2017; Longo & Zamudio, 2017). Moreover, amphibians tend to host skin communities that are unique to each host species, even when living in the same habitat and experiencing the same environmental conditions (McKenzie et al., 2012; Kueneman et al., 2014; Walke et al., 2014; Belden et al., 2015; Vences et al., 2016; Sabino-Pinto et al., 2017). Our understanding of the relative importance of fluctuation in other environmental variables, such as moisture, on skin-associated microbial communities remains poorly understood, especially for terrestrial ectotherms (Bird et al., 2018; but see Varela et al., 2018).

We sampled the skin of red-eye tree frog (*Agalychnis callidryas*) and hourglass frog (*Dendropsophus ebraccatus*), two of the most abundant, common and sympatric pond-breeding amphibian species in the Neotropics (Donnelly & Guyer, 1994) to examine how time scale and environmental conditions, specifically rainfall and temperature, affect their skin-associated bacterial communities. In this study, we: (1) described amphibian skin bacterial communities at three different time scales: years, seasons and days, (2) identified the bacterial taxa driving variation at different time scales, and (3) addressed potential links between temporal changes on skin bacterial communities and rainfall events or temperature shifts at the lowland tropical pond where samples were collected. By temporally assessing variation in host-associated microbial communities and identifying the environmental variables linked with such shifts, we can better understand factors that impact the structure of skin bacterial communities.

## Materials and Methods

### Study site and field survey

We collected skin samples of adult *A. callidryas* and *D. ebraccatus* at a single pond to examine long-term (annual and seasonal) and short-term (sampling day) variation in the skin bacterial communities. The study site, Ocelot Pond at Parque Nacional Soberanía, Panamá (50 m elevation), is a lowland, permanent, rain-fed pond. Ocelot Pond lies in seasonal moist forest characterized by a pronounced dry season from mid-December to mid-April/early May, and an extended wet season (Condit et al., 2001). The region receives approximately 2,700 mm of rain per year. On average, only about 300 mm of rain falls during the dry season. Temperatures vary relatively little throughout the year (average annual temperature = 26.3 ± 0.54 °C). We obtained daily average precipitation and air temperature measurements from the Physical Monitoring Program of the Smithsonian Tropical Research Institute (STRI).

We took one sample of skin bacterial communities from each individual of red-eyed treefrogs, *A. callidryas,* (*N* = 48) and hourglass treefrogs, *D. ebraccatus,* (*N* = 49) using sterile skin swabs as described in detail by Walke et al. (2015) and Belden et al. (2015). Swabs were placed individually in 1.5 ml sterile microcentrifuge tubes and were then stored at −80 °C until processing. Most animals were released on site immediately after sampling, except for individuals captured in 2013 (Table 1), which were used later in an experiment. Animal use was approved by the Institutional Animal Care and Use Committees of Virginia Tech (11-105-BIOL and 13-097-BIOL) and the Smithsonian Tropical Research Institute (2011-1110-2014 and 2013-0401-2016-A3), and was completed with permission from the Ministerio de Ambiente in Panamá (SE/A-47-12, SEX/A-65-12, SEX/A-77-12, SEX/A-89-12 and SEX/A-113-13).

**Table 1:** Sample sizes for field surveys assessing temporal differences in amphibian skin-associated bacterial communities on *Agalychnis callidryas* and *Dendropsophus ebraccatus*.

Sampling year	Sampling season	Sampling day	Time scale analysis	*Agalychnis callidryas*	*Dendropsophus ebraccatus*
2012	Wet	29-Aug-12	y	10	10
2013	Wet	14-Jun-13	y	10	10
2014	Wet	9-Jun-14	y/s/d		3
2014	Wet	27-Jul-14	y/s/d	6	2
2014	Wet	28-Jul-14	y/s/d	4	5
2014	Dry	21-Dec-14	s	3	
2014	Dry	22-Dec-14	s	4	
2014	Dry	30-Dec-14	s	2	
2015	Dry	11-Mar-15	s		4
2015	Dry	15-Mar-15	s		6
2015	Wet	28-Jun-15	y/s/d		9
2015	Wet	25-Jul-15	y/s/d	9	

**Note:**

Time scale analysis indicates when samples were used in analysis of variation sampling years (y), seasons (s) or days (d) for each treefrog species.

### DNA extraction and sequencing

DNA was extracted from swabs using the Qiagen DNeasy blood and tissue kit (Valencia, CA, USA), with an initial incubation step with lysozyme of 1 h at 37 °C. Relative abundance of operational taxonomic units (OTUs, which approximate bacterial species) based on 16S rRNA gene amplicon sequencing was used to assess bacterial community structure. For the community characterization, the V4 region of the 16S rRNA gene was amplified using the primers 515F and individually barcoded 806R (Caporaso et al., 2012). PCRs were run in triplicate, pooled and visualized on a 1% agarose gel, and quantified using a Qubit 2.0 fluorometer (Invitrogen, Carlsbad, CA, USA). These samples were included in seven different sequencing runs. For each run, PCR products (200 ng/sample) were pooled to make a composite sample and cleaned with the QIAquick PCR Purification Kit (Qiagen, Valencia, CA, USA). Final pooled samples were sent for sequencing on an Illumina MiSeq platform using the 250 bp paired-end (2013 samples) or single-end (2012, 2014 and 2015 samples) strategy at the Dana-Farber Cancer Institute of Harvard University.

Raw forward sequences from the Illumina 16S rRNA gene amplicon sequencing from the seven sequencing runs were processed and quality-filtered using the default parameters of the quantitative insights into microbial ecology pipeline (QIIME, v. 1.9.1; Caporaso et al., 2010a), with the exception that we allowed for no errors in the barcodes, the minimum Phred quality score (*q*) was 20, we increased the number of minimum consecutive low-quality base calls allowed before truncating a read (*r*) to 10, and decreased the fraction of the minimum number of consecutive high-quality base calls to include a read (*p*) to 0.5. Sequences were clustered into OTUs based on a 97% similarity threshold using the UCLUST method (Edgar, 2010), and representative OTUs were the most abundant sequence within the cluster. Representative sequences were aligned to the Greengenes 13_8 reference database (DeSantis et al., 2006) using PyNAST (Caporaso et al., 2010b) and assigned taxonomy using the RDP classifier (Wang et al., 2007). Prior to statistical analyses, we removed all OTUs with less than 0.01% of the total number of sequences (Bokulich et al., 2013), as well as all Archaea, chloroplast, and mitochondrial sequences. Then the dataset was rarefied at a depth of 11,500 reads/sample. The final dataset after rarefaction included 97 samples, with a range of 128–344 OTUs/sample.

### Data analysis and statistical methods

#### Overview

Temporal variation in bacterial community diversity (alpha diversity) and community structure (beta diversity) in *A. callidryas* and *D. ebraccatus* was evaluated at three time scales (Table 1). Larger temporal scales were assessed with annual and seasonal variation; one shorter temporal scale was assessed across sampling days. Annual variation was assessed using samples of *A. callidryas* and *D. ebraccatus* collected in June or July from 2012 to 2015 (*N* = 39 individuals per species total across the 4 years). Seasonal variation was assessed by comparing *A. callidryas* (*N* = 19) and *D. ebraccatus* (*N* = 19) sampled between the dry and wet seasons (*A. callidryas*: December 2014 and July 2014; *D. ebraccatus*: March 2015 and July 2015). Daily temporal variation was assessed with a subset of samples from 2014 to 2015 using individuals that were sampled within days of one another. If analyses indicated differences among years, we subsequently identified the OTUs driving those differences. We used indicator species analysis to identify the OTUs exclusively associated with a specific time point as well as those found across all frogs from each species at every sampling year (core microbiome). Finally, we examined associations between temporal variation in the skin microbiome and environmental variables (temperature and rainfall) using climate data obtained from the STRI. Amplicon sequences from the 2012 survey were deposited in the NCBI Sequence Read Archive (study accession number: SRP062596) as part of Belden et al. (2015), and sequences from 2013 were deposited in PRJNA504463. The remainder of the sequences was submitted as part of the present study in PRJNA504466. All statistical analyses were conducted in R v. 3.3.3 (R Development Core Team, 2017) using the vegan package (v. 2.4-5; Oksanen et al., 2017) unless noted otherwise.

#### Temporal variation in alpha and beta diversity on different time scales

Differences in alpha diversity across years, seasons and days were individually tested using OTU richness (estimated as the total number of OTUs per individual frog), Faith’s phylogenetic diversity and the Shannon index, which accounts for species abundance and evenness. These metrics were computed with QIIME 1.9 (Caporaso et al., 2010a) and were fitted to generalized linear models (GLMs) for each species with year, season and sampling day as fixed effects. GLMs were run separately for each temporal scale. OTU richness analyses were performed with a negative binomial error distribution (function glm.nb, package MASS, Venables & Ripley, 2002), while Faith’s phylogenetic diversity and the Shannon index were performed with a gamma error distribution (function glm, package lme4, Bates et al., 2015). The reported test statistic was Chi-Square (*X*^2^). Lastly, post hoc multiple comparisons of years and sampling days were conducted with Tukey tests (function glht, package multcomp, Hothorn, Bretz & Westfall, 2008).

Permutational multivariate analyses of variance (PERMANOVA, function adonis) and homogeneity of multivariate dispersion (PERMUTEST, function betadisper) were performed to determine if differences in bacterial community structure (location) and variability (dispersion) were explained by temporal variation. Pairwise comparisons using Tukey’s honest significance difference method were conducted on the betadisper distances. Bray–Curtis dissimilarities, based on OTU relative abundance data, were visualized with principal coordinate analysis. Results from the Jaccard dissimilarity distance matrix, which is based on OTU presence/absence, showed a similar pattern.

When PERMANOVA results suggested significant differences between skin-associated bacterial communities on frogs through time, indicator species analyses (function IndVal, package labdsv, Roberts, 2016) were conducted to identify bacterial taxa that showed exclusivity or fidelity to a year. Indicator OTUs were defined as a *P* < 0.05 and an indicator value of more than 0.7 (as in Becker et al., 2015). We controlled for the false discovery rate of multiple comparisons using the Benjamini & Hochberg (1995) method. Additionally, the core microbiome for each species, defined here as OTUs present on 100% of all the frogs across years, was calculated with QIIME 1.9.1 and was visualized with a heatmap (function heatmap.2, package gplots; Warnes et al., 2016).

#### Links between environmental variables and community composition

For our environmental variables of rainfall and temperature, we obtained daily average precipitation and air temperature measurements from the Barro Colorado Station at the STRI. For rainfall, we initially calculated the monthly and weekly averages and sums. To assess daily variation, additional rainfall metrics were calculated: the average and sum from 2 days prior to and on the day of sampling (as in Bletz et al., 2017); and the sum of day before, day of and day after collection. Daily rainfall is manually recorded the following morning; therefore, “day after” measurements were included to consider rainfall events during the night of sampling. Average temperatures were also calculated at the monthly, weekly and both daily scales. We were unable to include temperature calculations for two sampling days (27 and 28 of July 2014) due to instrument malfunctions. To use the most complete temperature data set, we tested for differences between average temperature for months, weeks and days. High collinearity between explanatory variables was anticipated (Zuur et al., 2009); therefore, to identify which specific environmental variables for rainfall had the highest explanatory power, we conducted a variable model selection based on the lowest Akaike’s information criterion (AIC) in canonical (constrained) correspondence analysis (CCA) (functions add1 and cca). Models that included variables with higher AIC were dropped until the best-fit model was selected (Gardener, 2014). The best-fit model resulted in us using the summation of day before, day of and day after sampling and the average monthly temperature as explanatory variables in subsequent models examining impacts on the skin bacterial communities.

To examine how rainfall and temperature affected community structure at each sampling day, we separately analyzed the skin bacterial communities of each treefrog species using a CCA, performed by the function cca (Ter Braak, 1986). We ran permutation tests on the overall CCA model (function anova) and on each variable (anova, by = “terms”) to test the significance of each environmental variable as a predictor of community composition. Only explanatory variables that explained variation in beta diversity are represented in the final results (function autoplot, package ggvegan, Simpson, 2017).

To further explore impacts of temporal variation on associations between bacterial community and environmental variables, we conducted a Mantel test (function mantel) using 1,000 permutations per each of the distance matrix pairs. Euclidean distance matrices were used for rainfall and temperature, and Bray–Curtis distances were used for bacterial community structure. Finally, we performed variation partitioning (Borcard, Legendre & Drapeau, 1992; Legendre, 2008) to assess how much variation in community structure could be attributed to rainfall and temperature independently and combined.

## Results

### Bacterial diversity and community structure varied at different time scales

**Figure 1:**
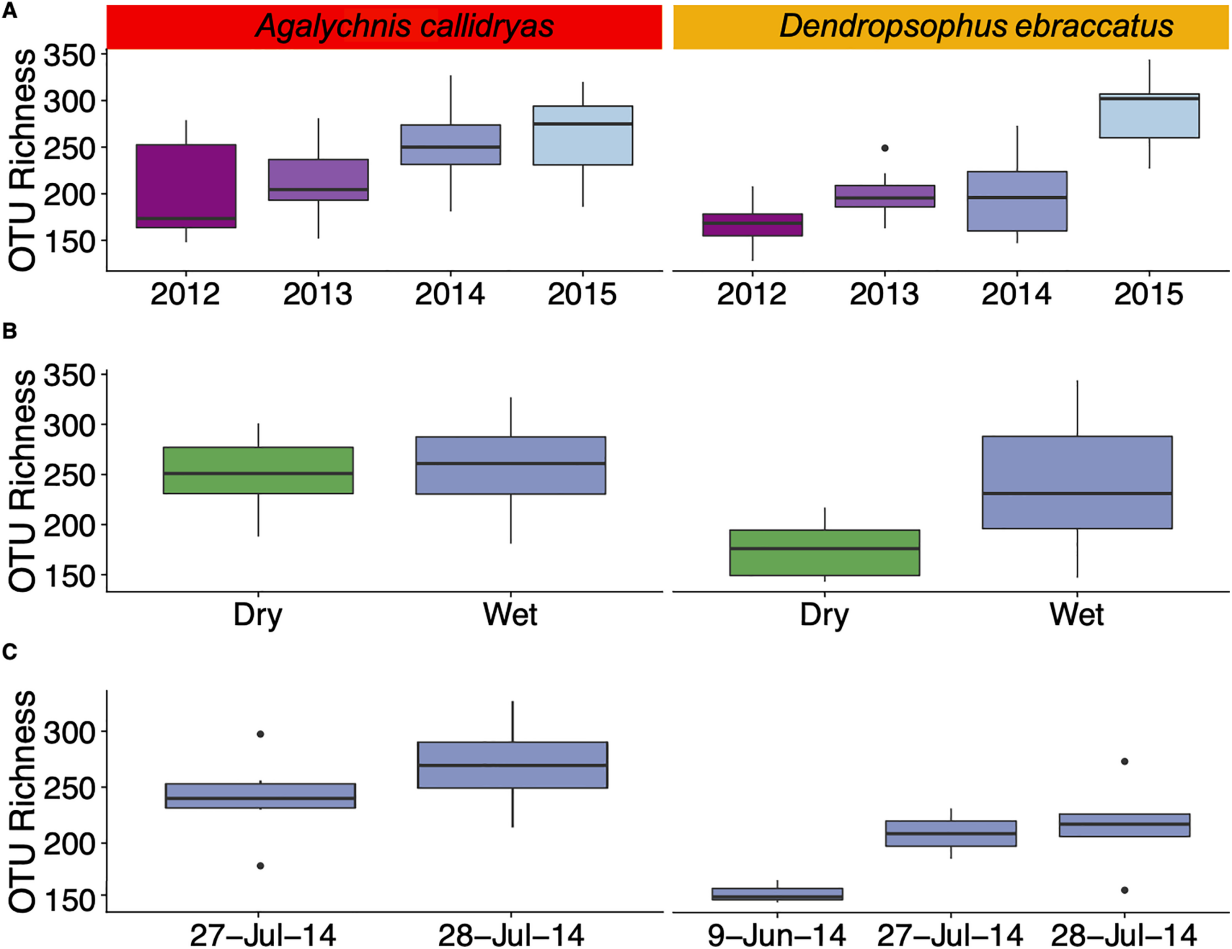
Alpha diversity (OTU Richness) of skin bacterial communities of *Agalychnis callidryas* and *Dendropsophus ebraccatus* at different temporal scales. Alpha diversity (OTU Richness) of skin bacterial communities of *Agalychnis callidryas* and *Dendropsophus ebraccatus* across sampling years (A), seasons (B) and days. (C) Annual values for OTU Richness include only the wet seasons of four consecutive years. Seasonal values include dry and wet seasons for 2014 and 2015. Daily values include only multiple sampling days in the wet season of 2014. Additional alpha diversity metrics are in the Supplementary Materials.

Bacterial community diversity (alpha diversity) on the skin of *A. callidryas* and *D. ebraccatus* varied through time at different scales. At large time scales, we found significant differences in OTU richness (ANOVA, *A. callidryas*: *X*^2^ = 13.31; *D. ebraccatus*: *X*^2^ = 30.37, both d*f* = 3, *P* < 0.01; Fig. 1A) and Faith’s phylogenetic diversity in both treefrog species across years (ANOVA, *A. callidryas*: *X*^2^ = 13.94; *D. ebraccatus*: *X*^2^ = 58.87, both d*f* = 3, *P* < 0.01; Fig. S1A). Community evenness (Shannon index) was not significantly different across years in *A. callidryas* (ANOVA, *X*^2^ = 6.25, d*f* = 3, *P* = 0.10; Fig. S2A), but was significantly different in *D. ebraccatus* (ANOVA, *X*^2^ = 17.67, d*f* = 3, *P* < 0.001; Fig. S2A). Generally, there was an overall increase in OTU richness and phylogenetic diversity from 2012 to 2015 in the skin microbiome of both frog species with the exception of 2013.

When examining seasonality in alpha diversity of bacterial communities we found differences between the dry and wet season for *D. ebraccatus*, but not for *A. callidryas*. Community diversity and evenness did not differ across seasons in *A. callidryas* (ANOVA, richness: *X*^2^ = 0.06, d*f* = 1, *P* = 0.81, Fig. 1B; Faith’s phylogenetic diversity: *X*^2^ = 0.01, d*f* = 1, *P* = 0.98, Fig. S1B; Shannon index: *X*^2^ = 0.57, d*f* = 1, *P* = 0.45, Fig. S2B), but did in *D. ebraccatus* (ANOVA, richness: *X*^2^ = 61.13, d*f* = 1, *P* < 0.001, Fig. 1B; Faith’s phylogenetic diversity: *X*^2^ = 24.18, d*f* = 1, *P* < 0.001, Fig. S1B; Shannon index: *X*^2^ = 3.56, d*f* = 1, *P* = 0.05, Fig. S2B).

At short time scales, comparisons of OTU richness across sampling days were significant for *D. ebraccatus* (ANOVA, *X*^2^ = 10.75, d*f* = 2, *P* = 0.005), but not for *A. callidryas (*ANOVA, *X*^2^ = 1.47, d*f* = 1, *P* = 0.22, Fig. 1C). We found no significant differences in Faith’s phylogenetic diversity and Shannon index across sampling days in both treefrog species (ANOVA, *A. callidryas*: *X*^2^ = 0.67 and 0.17, both d*f* = 1 and *P* > 0.05; *D. ebraccatus*: *X*^2^ = 3.66 and 0.02, both d*f* = 2, *P* > 0.05; Figs. S1C and S2C).

**Figure 2:**
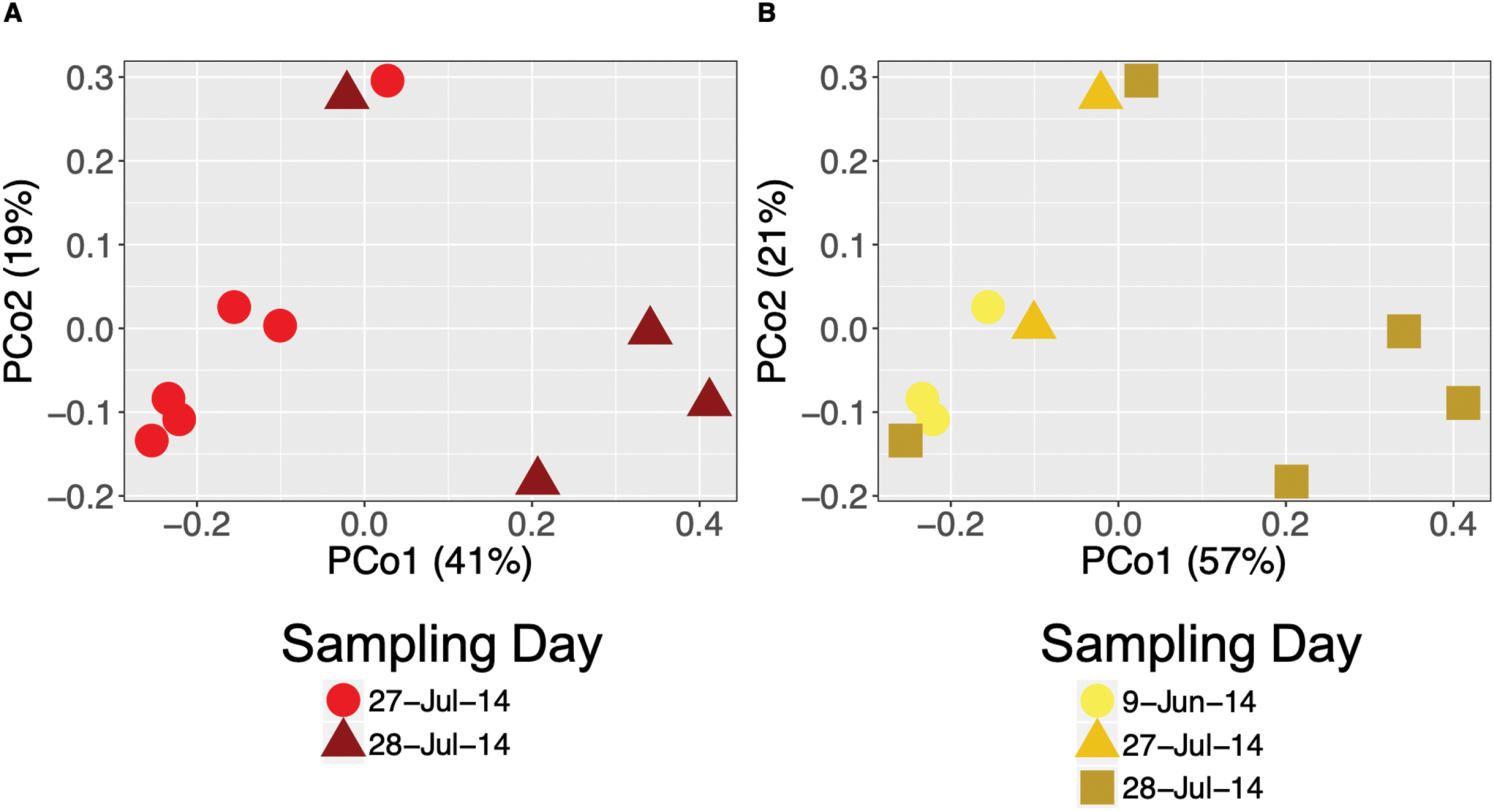
Daily variation of bacterial community structure on the skin of *Agalychnis callidryas* and *Dendropsophus ebraccatus*. Beta diversity of bacterial communities based on Bray–Curtis dissimilarity of *A. callidryas* (A) and *D. ebraccatus* (B) grouped by sampling day. Each point represents the skin bacterial community on a single individual.

We found temporal changes in bacterial community structure (beta diversity) based on Bray–Curtis dissimilarity distances at all time scales. Community structure differed across years (PERMANOVA for *A. callidryas:* Pseudo-*F* = 14.016, *R*^2^ = 0.53 and *D. ebraccatus:* Pseudo-*F* = 9.28, *R*^2^ = 0.44; both *P* < 0.001, Figs. S3A and S3B), seasons (PERMANOVA for *A. callidryas:* Pseudo-*F* = 2.19, *R*^2^ = 0.12, *P* = 0.05 and; *D. ebraccatus:* Pseudo-*F* = 5.48, *R*^2^ = 0.24, *P* = 0.0026; Figs. S3C and S3D) and sampling days (PERMANOVA, *A. callidryas:* Pseudo-*F* = 3.58, *R*^2^ = 0.31, *P* = 0.01; Fig. 2A and *D. ebraccatus:* Pseudo-*F* = 4.23, *R*^2^ = 0.55, *P* < 0.01; Fig. 2B) in both treefrog species. Dispersion from centroid across years was significantly different, with frogs in 2013 having communities more similar to one another (Permutest, *A. callidryas: F* = 26.696, *P* < 0.001 and *D. ebraccatus: F* = 27.402 *P* = 0.001). Dispersion between seasons was significantly different for *A. callidryas* (Permutest, *F* = 13.45, *P* = 0.005), but not for *D. ebraccatus* (Permutest, *F* = 0.3047, *P* = 0.58). Lastly, dispersion between sampling days was significant in *D. ebraccatus* (Permutest, *F* = 21.84, *P* = 0.001) but not in *A. callidryas* (Permutest, *F* = 4.51, *P* = 0.07).

### OTUs driving temporal variation

We identified a total of 540 OTUs in the skin of treefrogs and three phyla contributed 96% of the total relative abundance: Proteobacteria (63%), Actinobacteria (29%) and Firmicutes (4%). However, indicator species analysis identified a distinct set of OTUs associated with *A. callidryas* and *D. ebraccatus* across years with few OTUs shared between species (Table S1). By far, the highest number of indicator OTUs was recorded for samples from 2013 in both species: *A. callidryas* (*N* = 88) and *D. ebraccatus* (*N* = 75). Most indicator OTUs shared between treefrog species were in the phyla Proteobacteria (68%), including three OTUs associated with individuals of both frog species sampled in different seasons. We also found higher numbers of unique OTUs in both species in the dry season when comparing across seasons (*A. callidryas*: dry season *N* = 26/wet season *N* = 10; *D. ebraccatus* dry season *N* = 93/wet season *N* = 9).

**Figure 3:**
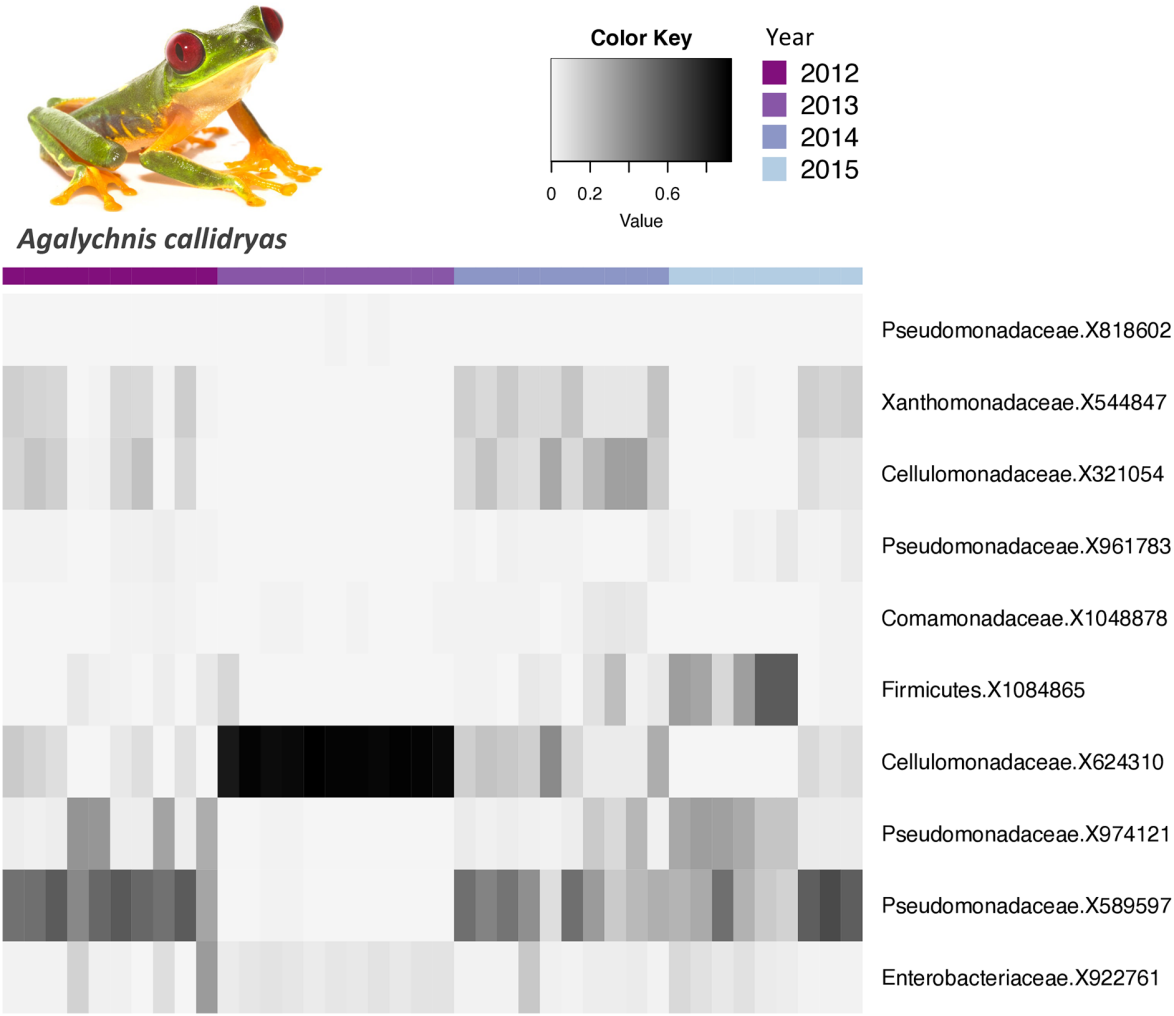
Relative abundance of core bacterial taxa on *Agalychnis callidryas* across years. Relative abundance of *Agalychnis callidryas* core bacterial taxa (OTUs found on 100% of the frogs) across years. Darker shades indicate higher relative abundances and lighter shades indicate lower relative abundances. Photo credit: Brian Gratwicke.

**Figure 4:**
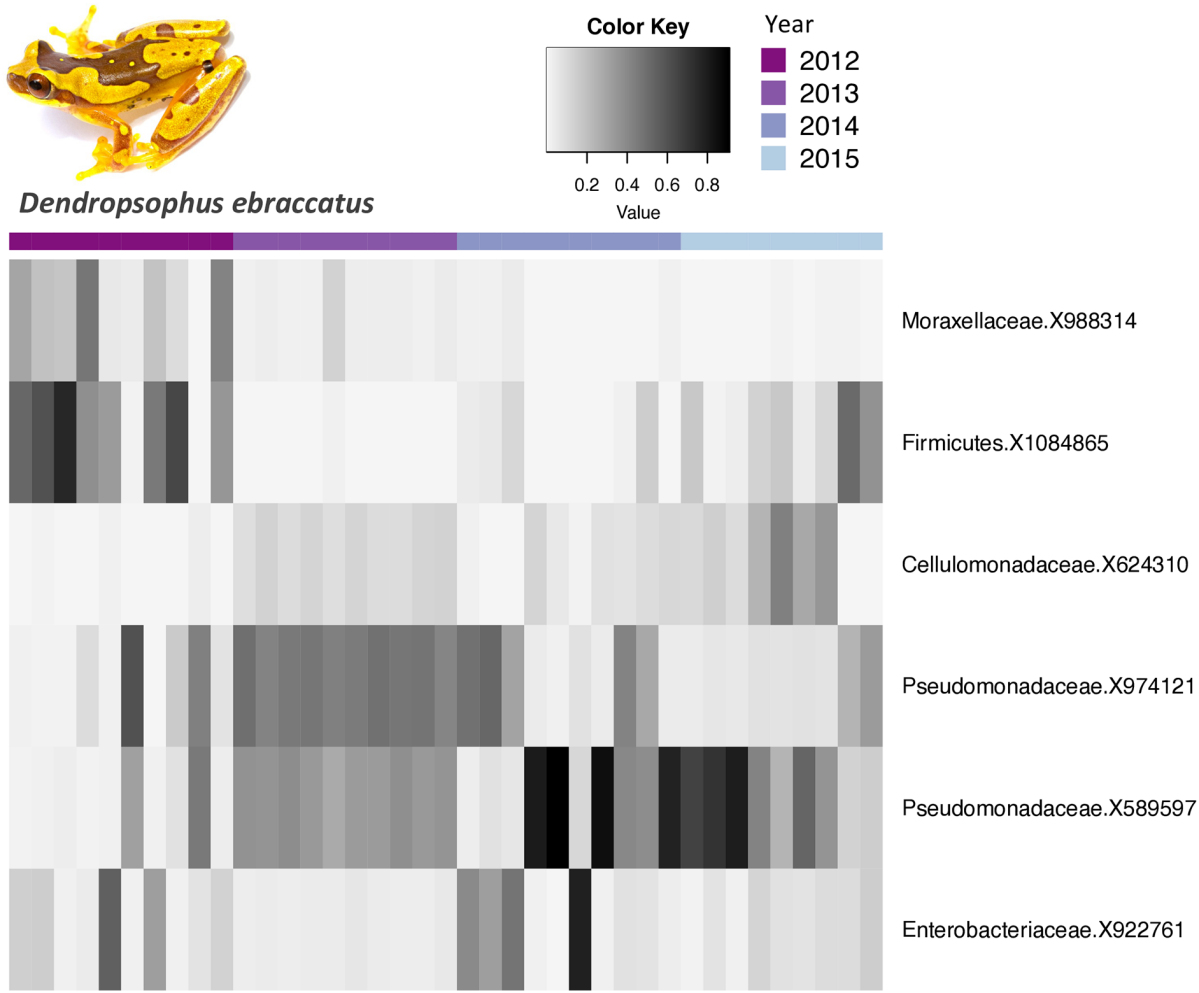
Relative abundance of core bacterial taxa on *Dendropsophus ebraccatus* across years. Relative abundance of *Dendropsophus ebraccatus* core bacterial taxa (OTUs found on 100% of the frogs) across years. Darker shades indicate higher relative abundances and lighter shades indicate lower relative abundances. Photo credit: Brian Gratwicke.

The core bacterial communities (OTUs present in 100% of the samples) for *A. callidryas* and *D. ebraccatus* was comprised of only 10 and six OTUs, respectively. Five of these OTUs were shared between both treefrog species. The vast majority of OTUs in the core community belonged to the phylum Proteobacteria (*A. callidryas* = 70% and *D. ebraccatus* = 67%). Moreover, all Proteobacteria belonged to the class Gammaproteobactera, including: Pseudomonadaceae, Xantomonadaceae, Commamonadaceae and Enterobacteriaceae. The relative abundance of the core OTUs varied considerably across individuals sampled at the same time point. Interestingly, individuals collected in 2013 had more similar abundances in their core communities, but strikingly differed from other years in both *A. callidryas* (Fig. 3) and *D. ebraccatus* (Fig. 4). Relative abundance of two core OTUs varied notably in 2013 compared to the other years in *A. callidryas:* Pseudomonadaceae X589596 (relative abundance, mean ± s.d. in 2012 = 50 ± 9%; 2013 = 0.5 ± 0.4%; 2014 = 34 ± 15%; 2015 = 37 ± 20%) and Cellumonadaceae X624310 (relative abundance, mean ± s.d. in 2012 = 6 ± 6%; 2013 = 89 ± 3%; 2014 = 16 ± 11%; 2015 = 3 ± 4%). Similar shifts in relative abundance across years were observed in *D. ebraccatus* for Pseudomonadaceae X9744121 (relative abundance, mean ± s.d. in 2012 = 14 ± 21%; 2013 = 46 ± 3%; 2014 = 22 ± 20%; 2015 = 11 ± 10%, Fig. 4). No core OTUs were identified by indicator species analysis.

### Rainfall and temperature are linked to temporal variation in skin-associated bacterial communities

**Figure 5:**
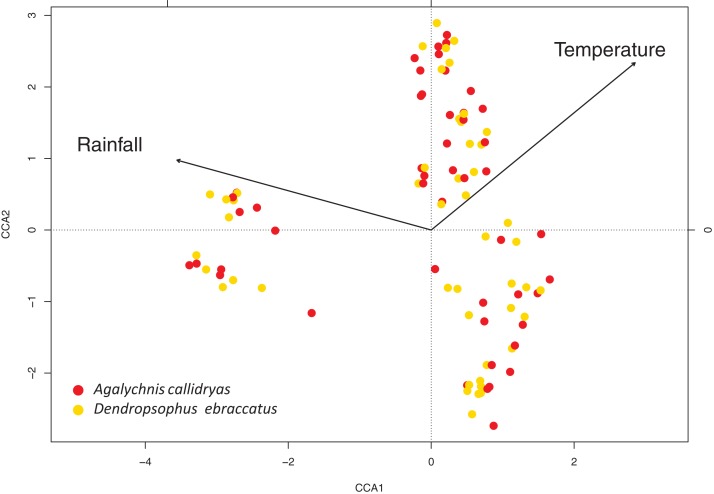
Canonical correspondence analysis of the variation in bacterial community structure on the skin of both treefrog species. Canonical (constrained) correspondence analysis shows that environmental variables (rainfall and temperature) are associated with temporal variation in community structure of skin-associated bacteria on *Agalychnis callidryas* (red) and *Dendropsophus ebraccatus* (yellow).

At short time scales, environmental variables (rainfall and temperature) were linked with bacterial community structure. In both treefrog species, we found significant correlations between the rainfall and temperature around the day of sampling and bacterial community structure (mantel test, *A. callidryas: R* = 0.5496; *D. ebraccatus: R* = 0.5097, both *P* < 0.001). Variance partitioning analysis showed that rainfall, calculated as the sum of day before, day of and day after sampling, explained most of the variation in community structure on both species. However, the proportion of variance explained for rainfall was larger in *A. callidryas* (21%) than in *D. ebraccatus* (9%). Temperature explained a smaller proportion of variation in *A. callidryas* (3%) and a similar proportion in *D. ebraccatus* (8%). Finally, most of the variance, 72% and 80%, respectively, was unexplained by the environmental variables measured in this study (Fig. 5).

## Discussion

We found clear temporal differences in skin bacterial communities on the skin of *A. callidryas* and *D. ebraccatus*. Time of sampling accounted for significant proportions of variation (years = 44–53%, seasons = 12–24% and days = 31–55%) observed in bacterial community diversity on both treefrog species. This, together with strong correlations found between community structure and rainfall and temperature on the day of sampling, emphasizes the role that rapidly changing environmental conditions may play on microbial community assembly and dynamics. Differences in response to shifts in environmental conditions among individuals and between species, despite cohabiting the same ponds, suggests that temporal dynamics observed in skin-associated bacterial communities could also be influenced by host-related factors and by biotic interactions among the bacteria.

The importance of temporal variability and time scale has been well established in human gut microbiome studies. Age is known to greatly alter microbial communities in the gut, where variation has been detected at time scales of months and years; however, changes in adult diets can result in parallel changes in the gut microbiome in just a few days (Gerber, 2014). Our study suggests that temporal instability of the skin bacterial communities through time might be largely regulated by external factors: abiotic conditions preceding sampling and the effects of these conditions on free-living microbial communities in the surrounding environment. This supports previous findings that amphibians, unlike humans (Costello et al., 2009; Oh et al., 2016), might continuously reacquire new microbes for their skin from the surrounding environments (Loudon et al., 2014). Although our study did not allow for repeated samples of individuals and did not have a large sample size at finer temporal scales, our results highlight variation in both alpha diversity metrics and community structure across sampling years, seasons and days.

In our study, the differences observed in bacterial communities through time were mainly associated with shifts in relative abundance of core taxa and indicator OTUs, associated with each sampling point. Both core and indicator OTUs were dominated by the phyla Proteobacteria, Actinobacteria, Firmicutes, Bacteroides and Verrucomicrobia. These major phyla also contribute key OTUs in the skin bacterial communities of other tropical amphibians (Longo et al., 2015; Hughey et al., 2017), including amphibians from the same region in eastern Panama (Belden et al., 2015; Rebollar et al., 2016). Other recent studies are consistent with this finding that changes in the relative abundance of key bacterial OTUs are mainly responsible for the temporal patterns observed in amphibian skin bacterial communities (Bletz et al., 2017; Sabino-Pinto et al., 2017). However, most studies on temporal variation of amphibian skin bacterial communities have focused on describing community shifts between different life stages (Kueneman et al., 2014; Longo et al., 2015; Bletz et al., 2017; Sabino-Pinto et al., 2017; Muletz-Wolz et al., 2018). Consequently, the microbes associated with changes in community composition are most likely acquired from environmental sources corresponding to various life stages (Prest et al., 2018). In addition, differences in the skin itself across life stages likely selects for different bacterial communities. For our study, only terrestrial adult stages were sampled, and variation in skin communities through time were not expected to be explained solely by differences in age nor environmental sources of bacteria (Walke et al., 2014; Rebollar et al., 2016; Varela et al., 2018).

We found that rainfall and temperature immediately preceding the day of sampling were strongly correlated with bacterial community structure on both treefrog species. In fact, rainfall alone explained as much as 21% of the temporal variation observed on *A. callidryas*. Although temperature can have a strong effect on community diversity and composition of host-associated microbiomes (Carrier & Reitzel, 2017), including amphibians (Kohl & Yahn, 2016), and free-living microbial communities (Pettersson & Bååth, 2003), we found that rainfall best explained observed shifts in the skin bacterial community of our study species. Our study site, Ocelot Pond, like other tropical lowland ecosystems, is characterized by little intra-annual variation in temperature (Whitfield et al., 2012) and high seasonality of precipitation (Condit et al., 2001). Moreover, different microhabitats around pond edges reduce ambient temperature variability in the canopy of lowland tropical forest (Scheffers et al., 2014); thus, arboreal amphibian hosts and their associated bacteria might be less exposed to temperature fluctuations. Rainfall events, however, are infrequent during the dry season, but short-term fluctuations in rainfall are common during the rainy season (Condit et al., 2001). Bacterial community diversity and evenness in both treefrogs increased across years from samples collected in 2012–2015. Likewise, rainfall generally decreased through time, with 2015 having the lowest rainfall recorded. Changes in environmental conditions in amphibian microhabitat have been previously linked to variation in diversity and composition of skin bacterial communities on temperate amphibians (Bletz et al., 2017; Kueneman et al., 2014; Longo et al., 2015). It has been proposed that changing environmental conditions and bacterial reservoirs can induce temporal shifts in amphibian skin bacterial communities (Belden & Harris, 2007; Loudon et al., 2014, 2016). For example, cold temperatures increased OTU richness in adult *Lithobates yavapaiensis* during the winter months (Longo et al., 2015), and fluctuations in OTU relative abundances on newt skin were linked to water temperature (Bletz et al., 2017). However, in these studies water temperatures have extreme seasonal fluctuations that may exceed the physiological tolerance levels for some symbionts (Erwin et al., 2015), and therefore, the conditions are quite different than in the tropical system we studied.

Interestingly, the dynamics of the amphibian fungal pathogen, *Batrachochytrium dendrobatidis*, have also been associated with patterns of rainfall and temperature in the Neotropics (James et al., 2015). Panamanian amphibian communities at higher elevations have been particularly hard hit by the spread of Bd, with some communities experiencing loss of >50% of species (Lips, 1999; Lips, Reeve & Witters, 2003; Lips et al., 2006). In our study, we examined amphibian species that are not highly susceptible to Bd, so that we could focus on non-pathogen induced changes in the skin bacterial communities. However, it could be important to ultimately examine links between these rapid changes we observed in OTU relative abundances and Bd infection dynamics in more susceptible host species. Overall, little is known about the functional significance of varying OTU relative abundances on amphibian skin bacterial communities. We have found that different bacterial communities can produced secondary metabolite profiles that are quite similar, which suggests that function may not be closely linked to structure in at least some skin bacterial communities (Belden et al., 2015). Our present data do not allow us to determine the functional outcome of changing OTU relative abundances through time, but we do provide evidence that small fluctuations in environmental conditions at time of sampling can have an important and rapid effect on OTU relative abundances, and these could potentially impact the dynamics of Bd.

Our observations revealed two specific patterns that might suggest the broader importance of environmental conditions on bacterial communities. First, there were significant seasonal effects in *D. ebraccatus,* and not in *A. callidryas*. This pattern suggests that different processes influenced the skin bacterial communities of each species. Specifically, community diversity and evenness were significantly higher in the wet season of 2015 on *D. ebraccatus*. Interestingly, the 2015 wet season was characterized by extended dry periods typical of the El Niño-Southern Oscillation cycle in Panamá. Since there was little rainfall for both dry and wet seasons of 2015, we would expect to see similar temporal microbiome responses. However, some individuals of *D. ebraccatus,* but none of *A. callidryas,* were found closer to or partially submerged in the water during the wet season of 2015 (A. Estrada, 2015, personal observation) potentially allowing bacterial colonization from a different environmental source. While *A. callidryas* and *D. ebraccatus* adults live in the forest canopy (Ibáñez, Rand & Jaramillo, 1999; Duellman, 2001), *D. ebraccatus* can change their reproductive mode based on microhabitat conditions, and lay eggs in either aquatic or terrestrial substrates (Touchon & Warkentin, 2009), while *A. callidryas* lays only on vegetation. Environmental conditions that impact variation in behavior and microhabitat use between these two sympatric species might also drive differences in skin bacterial communities. Second, samples from 2013 displayed less variation among individual frogs and greater difference in alpha and beta community diversity in comparison to other years. Interestingly, environmental conditions around the day of sampling in 2013 had two of the most extreme values for rainfall (high) and temperature (low) reported for the duration of our study. Frogs sampled in 2013 were likely exposed to an intense short-term rain event, which could have washed-in or altered (Elmir et al., 2007) the bacterial communities of both species. Taken together, these results emphasize the role that abiotic factors play in natural variation of bacterial communities associated with amphibian skin.

Although shifts in amphibian skin bacterial diversity and relative abundance have recently been linked to extreme weather events (Familiar-López et al., 2017), the effect of large-scale climate fluctuations on bacterial symbionts is not well understood. The potential for microbes to influence amphibians’ fitness could change our current understanding of how both aquatic and terrestrial organisms adapt to global climate change. Overall, our results suggest that short-term amphibian microbiome studies may not be sufficient for finding clear patterns and making predictions about the community’s response to such large-scale events. Thus, an assessment of the effects of large-scale climate variability on microbiome stability and function will require long-term surveys and high-resolution environmental variables. Research on marine organisms also proposes that the host-microbe relationship is altered by fluctuations in environmental conditions (reviewed by Apprill, 2017). Most notably, the increase of ocean temperatures may alter the temporal stability of microbial communities of both of these marine taxa. Coral bleaching, the most visible consequence of microbiome dysbiosis, occurs after long-term, yet small, increases in seawater temperature (Brown, 1997). Although long-term monitoring of microclimatic conditions will be challenging, future work targeting long-term microbiome studies and their temporal fluctuations are necessary. Moreover, manipulative experiments involving precipitation changes (Beier et al., 2012) will provide us with a better understanding of the direct effects of rainfall and temperature on structural and functional stability of the amphibian skin-associated microbial communities under changing environmental conditions.

## Conclusions

Our study aimed to examine natural temporal dynamics of skin-associated bacterial communities of two common Neotropical treefrog species. By exploring the effect of large- and small-temporal scales and accounting for short-term environmental heterogeneity, we observed that variation detected daily, seasonally and annually were mainly explained by short-term rainfall events. Our findings suggest some caution should be taken in interpreting results from one sampling period, since these results cannot capture variation based on time scale and variation in local short-term environmental conditions. Considering natural variation in the microbiome could be important for studies of host-microbiome interactions.

## Supplemental Information

10.7717/peerj.7044/supp-1 Supplemental Information 1 Indicator OTUs associated with different years shared between *Agalychnis callidryas* and *Dendropsophus ebraccatus*. The indicator value index is a measure of the association between an OTU and a year, and ranges from 0 to 1, where a value closer to 1 implies a relatively stronger association. The P-values are corrected with multiple comparisons using the Benjamini & Hochberg method (1995).

10.7717/peerj.7044/supp-2 Supplemental Information 2 Alpha diversity (Faith’s phylogenetic diversity) of skin bacterial communities on *Agalychnis callidryas* and *Dendropsophus ebraccatus* at different temporal scales. Alpha diversity (Faith’s phylogenetic diversity) of skin bacterial communities on *Agalychnis callidryas* and *Dendropsophus ebraccatus* across sampling years (A), seasons (B) and days (C). Annual values for phylogenetic diversity include only the wet seasons of four consecutive years. Seasonal values include dry and wet seasons for 2014 and 2015. Daily values include only multiple sampling days in the wet season of 2014.

10.7717/peerj.7044/supp-3 Supplemental Information 3 Community evenness (Shannon) of skin bacterial communities on *Agalychnis callidryas* and *Dendropsophus ebraccatus* at different temporal scales. Community evenness (Shannon) of skin bacterial communities on *Agalychnis callidryas* and *Dendropsophus ebraccatus* across sampling years (A), seasons (B) and days (C). Annual values for evenness include only the wet seasons of four consecutive years. Seasonal values include dry and wet seasons for 2014 and 2015. Daily values include only multiple sampling days in the wet season of 2014.

10.7717/peerj.7044/supp-4 Supplemental Information 4 Annual and seasonal variation of bacterial community structure on the skin of *Agalychnis* *callidryas* and *Dendropsophus ebraccatus*. Beta diversity of bacterial communities based on Bray-Curtis dissimilarity of *A. callidryas* (left column) and *D. ebraccatus* (right column) grouped by year (AB) and season (CD). Each point represents the skin bacterial community on a single individual.

## Additional Information and Declarations

### Field Study Permissions

The following information was supplied relating to field study approvals (i.e., approving body and any reference numbers): Scientific collection permits were provided by the Panamanian authorities (Autoridad Nacional del Ambiente, now Ministerio de Ambiente) (SE/A-47-12, SEX/A-65-12, SEX/A-77-12, SEX/A-89-12 and SEX/A-113-13).

### Data Availability

The following information was supplied regarding data availability: Data is available at the National Center for Biotechnology Information (NCBI), accession numbers: PRJNA504463 and PRJNA504466.
