# Supplementary figures and images for: Skin bacterial communities of neotropical treefrogs vary with local environmental conditions at the time of sampling
Source: PeerJ. 2019 Jun 21;7:e7044. doi: 10.7717/peerj.7044 (PMC6590418; doi:10.7717/peerj.7044)

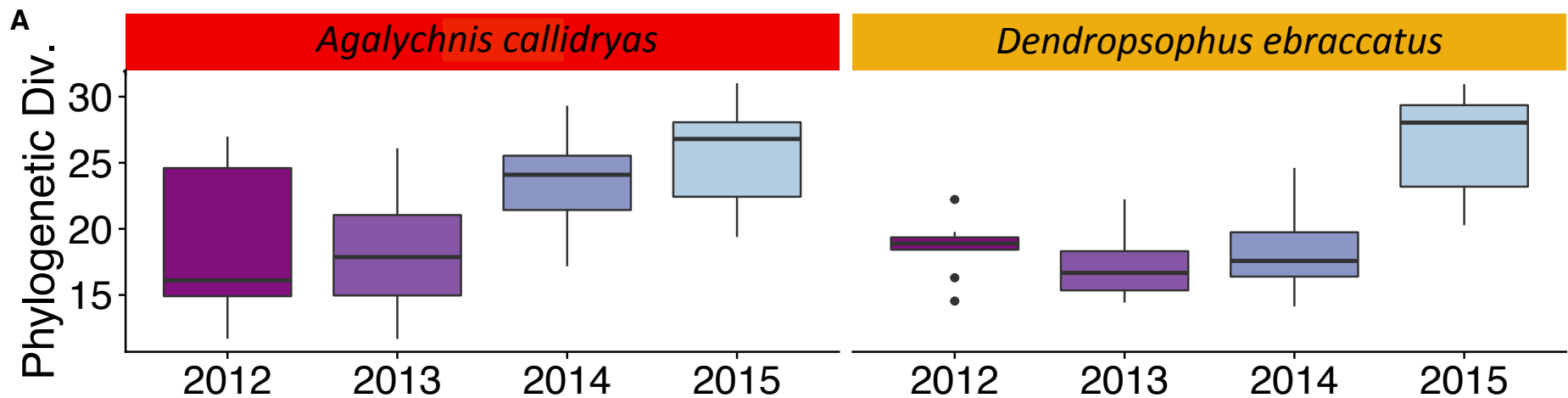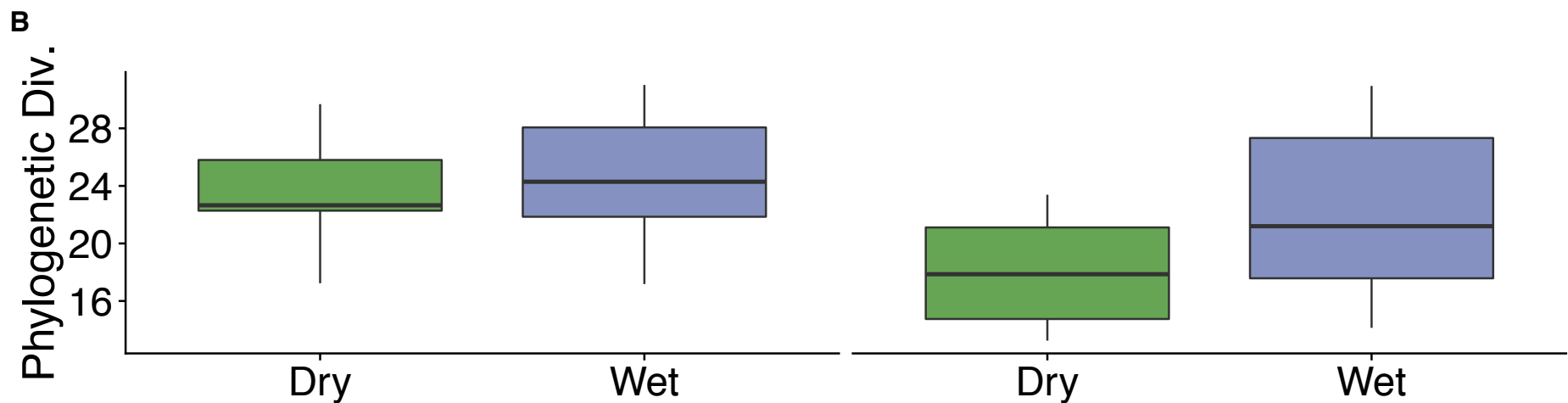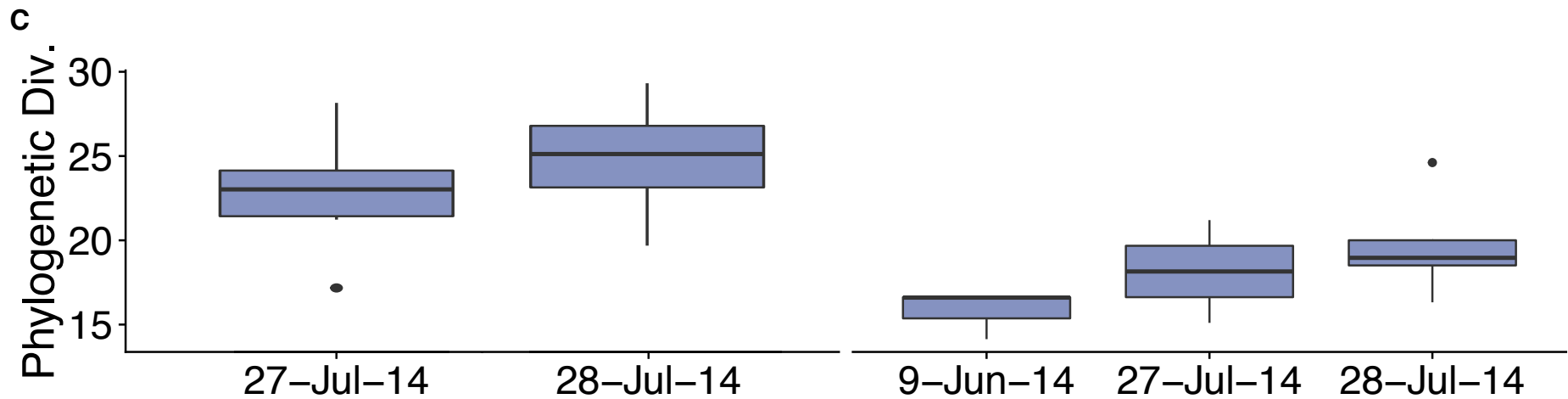

Supplement: Supplemental Information 2 — Alpha diversity (Faith’s phylogenetic diversity) of skin bacterial communities on Agalychnis callidryas and Dendropsophus ebraccatus across sampling years (A), seasons (B) and days (C). Annual values for phylogenetic diversity include only the wet seasons of four consecutive years. Seasonal values include dry and wet seasons for 2014 and 2015. Daily values include only multiple sampling days in the wet season of 2014. [file peerj-07-7044-s002.pdf]

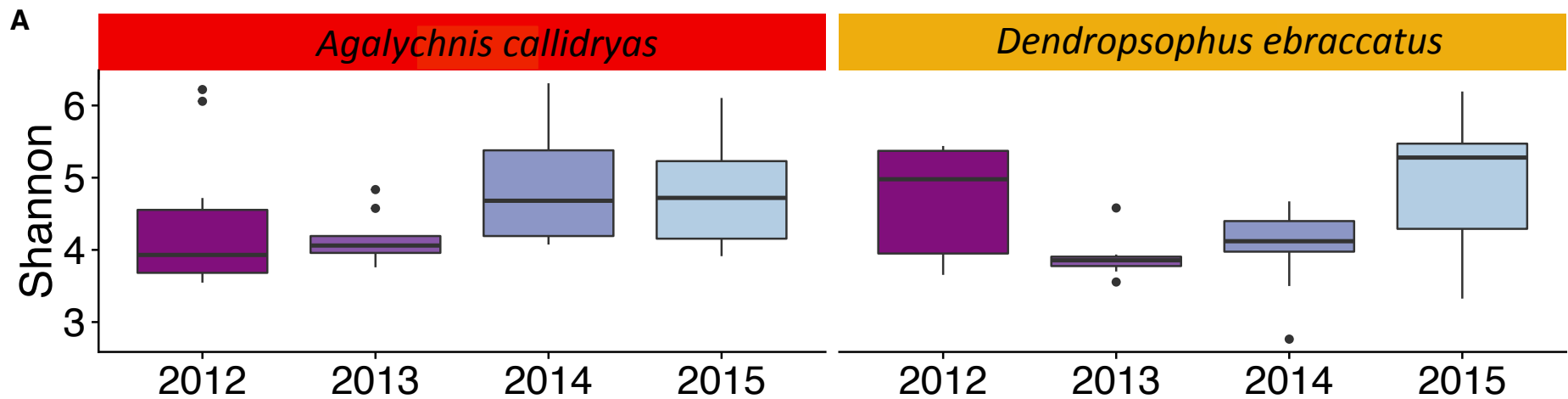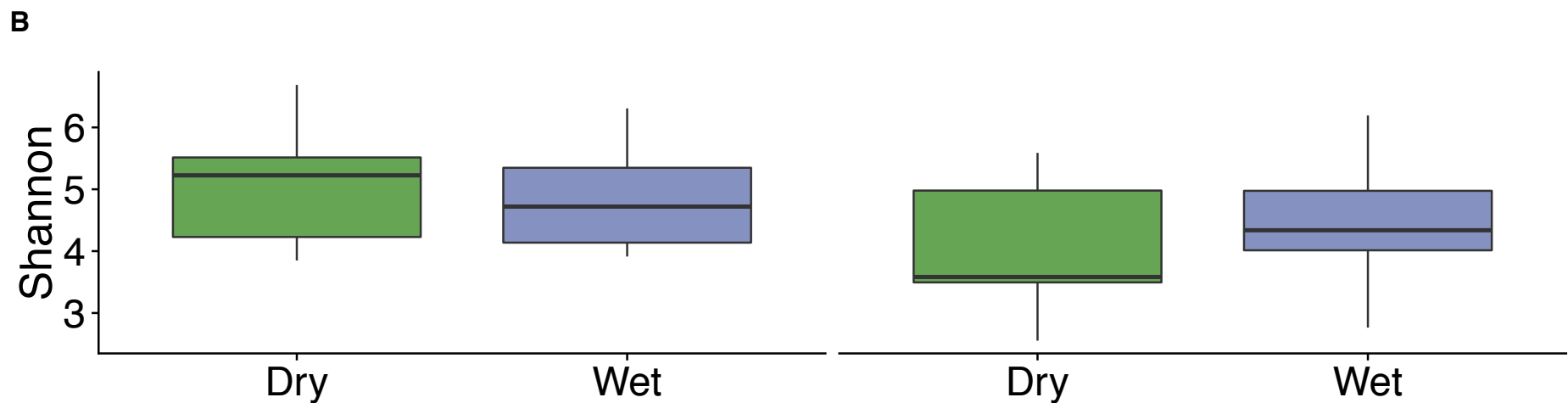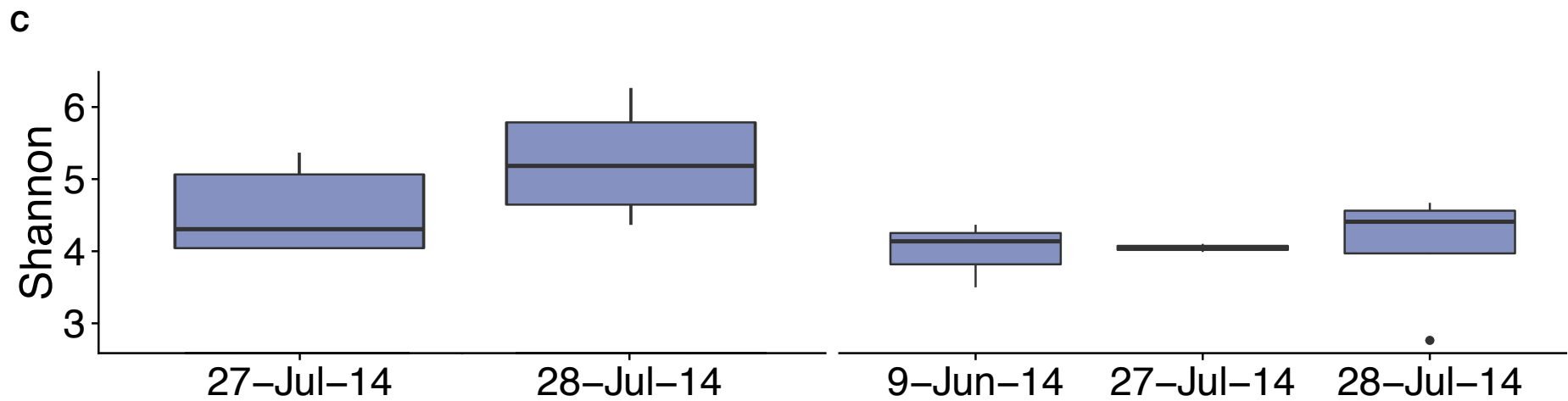

Supplement: Supplemental Information 3 — Community evenness (Shannon) of skin bacterial communities on Agalychnis callidryas and Dendropsophus ebraccatus across sampling years (A), seasons (B) and days (C). Annual values for evenness include only the wet seasons of four consecutive years. Seasonal values include dry and wet seasons for 2014 and 2015. Daily values include only multiple sampling days in the wet season of 2014. [file peerj-07-7044-s003.pdf]

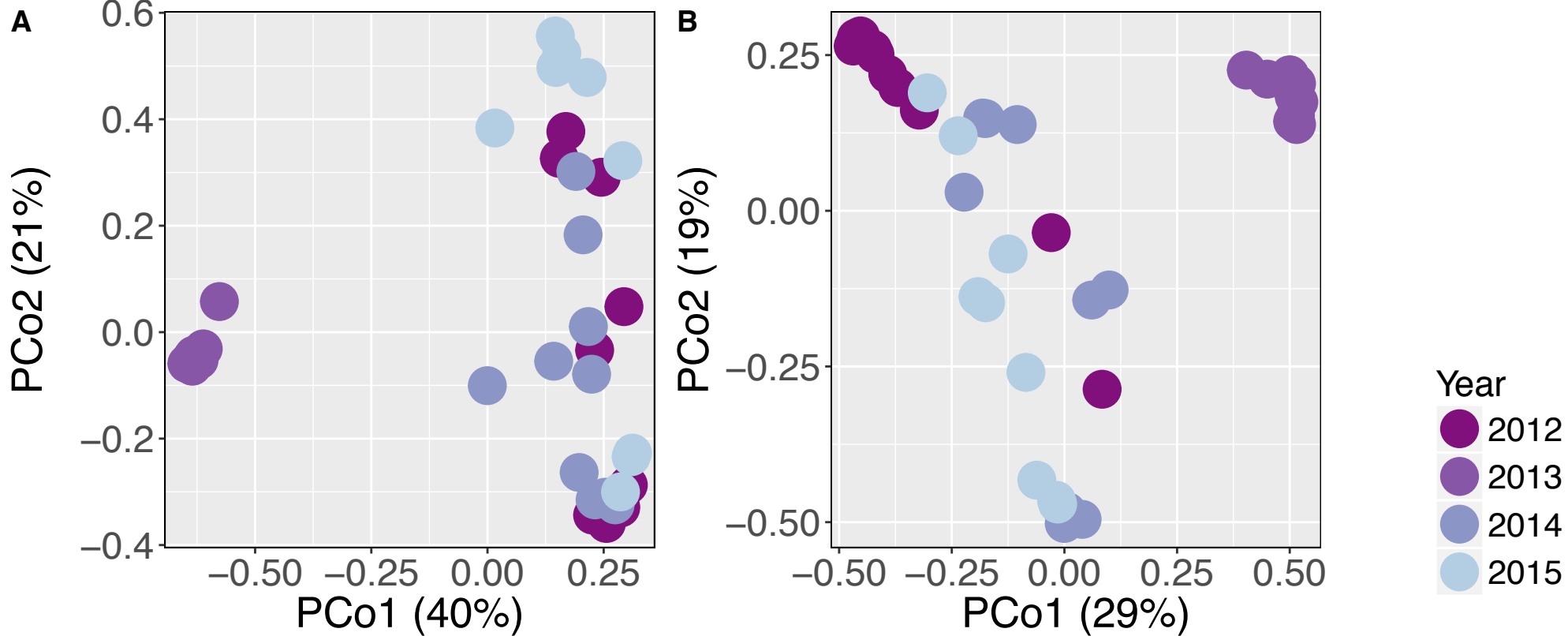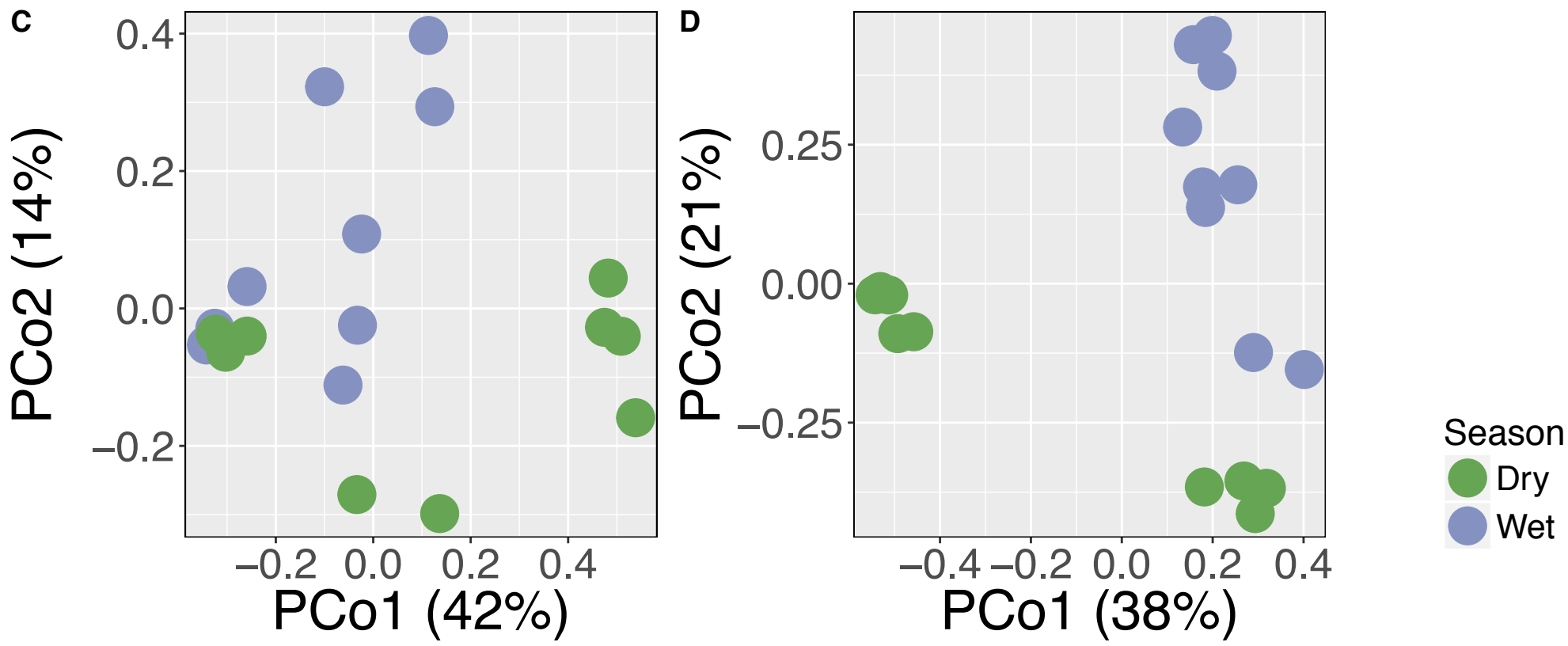

Supplement: Supplemental Information 4 — Beta diversity of bacterial communities based on Bray-Curtis dissimilarity of A. callidryas (left column) and D. ebraccatus (right column) grouped by year (AB) and season (CD). Each point represents the skin bacterial community on a single individual. [file peerj-07-7044-s004.pdf]
