# Supplementary material for: Skin bacterial communities of neotropical treefrogs vary with local environmental conditions at the time of sampling
Source: PeerJ. 2019 Jun 21;7:e7044. doi: 10.7717/peerj.7044 (PMC6590418; doi:10.7717/peerj.7044)
Supplement: Supplementary file 2 [file peerj-07-7044-s001.docx]

							Agalychnis callidryas		*Dendropsophus ebraccatus*	
**Sampling year**	**OTU ID**	**Phylum**	**Class**	**Order**	**Family**	**Genus**	**Indicator value index**	**P-value**	**Indicator value index**	**P-value**
2012	denovo70491	Verrucomicrobia	Verrucomicrobiae	Verrucomicrobiales	Verrucomicrobiaceae		0.881	0.003	0.626	0.036
2013	X578701	Actinobacteria	Actinobacteria	Actinomycetales	Nocardiaceae	Rhodococcus	0.972	0.003	0.834	0.003
2013	X4470206	Proteobacteria	Gammaproteobacteria	Pseudomonadales	Pseudomonadaceae	Pseudomonas	1.000	0.003	0.837	0.003
2013	X4434190	Proteobacteria	Gammaproteobacteria	Pseudomonadales	Pseudomonadaceae	Pseudomonas	1.000	0.003	0.837	0.003
2013	X544313	Proteobacteria	Gammaproteobacteria	Pseudomonadales	Pseudomonadaceae	Pseudomonas	0.984	0.003	0.844	0.003
2013	denovo997	Actinobacteria	Actinobacteria	Actinomycetales	Cellulomonadaceae	Cellulomonas	1.000	0.003	0.949	0.003
2013	X566578	Proteobacteria	Betaproteobacteria	Burkholderiales	Oxalobacteraceae		0.934	0.003	0.949	0.003
2013	X915948	Actinobacteria	Actinobacteria	Actinomycetales	Nocardiaceae	Rhodococcus	1.000	0.003	0.957	0.003
2013	X1083508	Proteobacteria	Gammaproteobacteria	Xanthomonadales	Xanthomonadaceae	Stenotrophomonas	0.999	0.003	0.966	0.003
2013	denovo291070	Proteobacteria	Gammaproteobacteria	Xanthomonadales	Xanthomonadaceae		0.855	0.003	0.973	0.003
2013	X536008	Proteobacteria	Gammaproteobacteria	Xanthomonadales	Xanthomonadaceae		0.954	0.003	0.975	0.003
2013	denovo298942	Actinobacteria	Actinobacteria	Actinomycetales	Cellulomonadaceae	Cellulomonas	0.845	0.003	0.982	0.003
2013	X1109607	Actinobacteria	Actinobacteria	Actinomycetales	Cellulomonadaceae	Cellulomonas	0.940	0.003	0.984	0.003
2013	X528421	Proteobacteria	Gammaproteobacteria	Enterobacteriales	Enterobacteriaceae	Citrobacter	0.789	0.003	0.988	0.003
2013	X750016	Bacteroidetes	Flavobacteriia	Flavobacteriales	[Weeksellaceae]	Chryseobacterium	0.992	0.003	0.991	0.003
2013	denovo118630	Actinobacteria	Actinobacteria	Actinomycetales	Cellulomonadaceae	Cellulomonas	0.923	0.003	0.991	0.003
2013	X558264	Proteobacteria	Betaproteobacteria	Burkholderiales	Alcaligenaceae	Achromobacter	1.000	0.003	0.994	0.003
2013	X969805	Proteobacteria	Alphaproteobacteria	Rhizobiales	Rhizobiaceae	Agrobacterium	0.932	0.003	0.996	0.003
2013	X429838	Actinobacteria	Actinobacteria	Actinomycetales	Cellulomonadaceae	Cellulomonas	1.000	0.003	1.000	0.003
2013	denovo298806	Actinobacteria	Actinobacteria	Actinomycetales	Cellulomonadaceae	Cellulomonas	0.894	0.003	1.000	0.003
2013	X323068	Actinobacteria	Actinobacteria	Actinomycetales	Cellulomonadaceae	Cellulomonas	0.949	0.003	1.000	0.003
2013	denovo49008	Actinobacteria	Actinobacteria	Actinomycetales	Cellulomonadaceae	Cellulomonas	0.993	0.003	1.000	0.003
2013	X592636	Proteobacteria	Alphaproteobacteria	Rhizobiales	Brucellaceae	Ochrobactrum	1.000	0.003	1.000	0.003
2013	X451696	Proteobacteria	Alphaproteobacteria	Rhizobiales	Brucellaceae	Ochrobactrum	1.000	0.003	1.000	0.003
2013	X646549	Proteobacteria	Gammaproteobacteria	Pseudomonadales	Pseudomonadaceae	Pseudomonas	0.970	0.008	0.964	0.003
2013	X570350	Proteobacteria	Alphaproteobacteria	Sphingomonadales	Sphingomonadaceae	Sphingobium	0.707	0.010	0.775	0.003
2013	X895220	Proteobacteria	Betaproteobacteria	Burkholderiales	Comamonadaceae		0.671	0.014	0.992	0.003
2013	X591708	Proteobacteria	Gammaproteobacteria	Xanthomonadales	Xanthomonadaceae	Stenotrophomonas	0.640	0.021	0.996	0.003
2013	denovo341643	Actinobacteria	Actinobacteria	Actinomycetales	Cellulomonadaceae	Cellulomonas	1.000	0.003	0.775	0.005
2013	X1060517	Proteobacteria	Betaproteobacteria	Burkholderiales	Oxalobacteraceae	Janthinobacterium	0.697	0.005	0.699	0.014
2014	denovo42701	Bacteroidetes	Flavobacteriia	Flavobacteriales	[Weeksellaceae]	Chryseobacterium	0.775	0.003	0.774	0.003
2014	denovo288776	Proteobacteria	Gammaproteobacteria	Oceanospirillales	Alcanivoracaceae	Alcanivorax dieselolei	0.992	0.003	0.981	0.003
2014	X793461	Proteobacteria	Gammaproteobacteria	Oceanospirillales	Alcanivoracaceae	Alcanivorax dieselolei	0.968	0.003	0.986	0.003
2015	X767063	Bacteroidetes	[Saprospirae]	[Saprospirales]	Chitinophagaceae	Chitinophaga	0.789	0.003	0.735	0.003
2015	X786876	Bacteroidetes	Flavobacteriia	Flavobacteriales	[Weeksellaceae]	Elizabethkingia meningoseptica	0.941	0.003	0.697	0.005
2015	X1042671	Bacteroidetes	[Saprospirae]	[Saprospirales]	Chitinophagaceae		0.792	0.016	0.726	0.005
2015	X527033	Verrucomicrobia	Verrucomicrobiae	Verrucomicrobiales	Verrucomicrobiaceae		0.662	0.021	0.650	0.009
2015	X377546	Proteobacteria	Alphaproteobacteria	Caulobacterales	Caulobacteraceae	Mycoplana	0.759	0.055	0.703	0.019
